# Oncolytic Virotherapy for Cancer: Clinical Experience

**DOI:** 10.3390/biomedicines9040419

**Published:** 2021-04-13

**Authors:** Shyambabu Chaurasiya, Yuman Fong, Susanne G. Warner

**Affiliations:** Department of Surgery, City of Hope National Medical Center, Duarte, CA 91010, USA; yfong@coh.org (Y.F.); suwarner@coh.org (S.G.W.)

**Keywords:** oncolytic virus, clinical trials, adenovirus, vaccinia virus, herpes virus, reovirus

## Abstract

Oncolytic viruses are a new class of therapeutics which are largely in the experimental stage, with just one virus approved by the FDA thus far. While the concept of oncolytic virotherapy is not new, advancements in the fields of molecular biology and virology have renewed the interest in using viruses as oncolytic agents. Backed by robust preclinical data, many oncolytic viruses have entered clinical trials. Oncolytic viruses that have completed some levels of clinical trials or are currently undergoing clinical trials are mostly genetically engineered viruses, with the exception of some RNA viruses. Reolysin, an unmodified RNA virus is clinically the most advanced oncolytic RNA virus that has completed different phases of clinical trials. Other oncolytic viruses that have been studied in clinical trials are mostly DNA viruses that belong to one of the three families: herpesviridae, poxviridae or adenoviridae. In this review work we discuss recent clinical studies with oncolytic viruses, especially herpesvirus, poxvirus, adenovirus and reovirus. In summary, the oncolytic viruses tested so far are well tolerated, even in immune-suppressed patients. For most oncolytic viruses, mild and acceptable toxicities are seen at the currently defined highest feasible doses. However, anti-tumor efficacies of oncolytic viruses have been modest, especially when used as monotherapy. Therefore, the potency of oncolytic viruses needs to be enhanced for more oncolytic viruses to hit the clinic. Aiming to achieve higher therapeutic benefits, oncolytic viruses are currently being studied in combination with other therapies. Here we discuss the currently available clinical data on oncolytic viruses, either as monotherapy or in combination with other treatments.

## 1. Introduction

**Figure 1:**
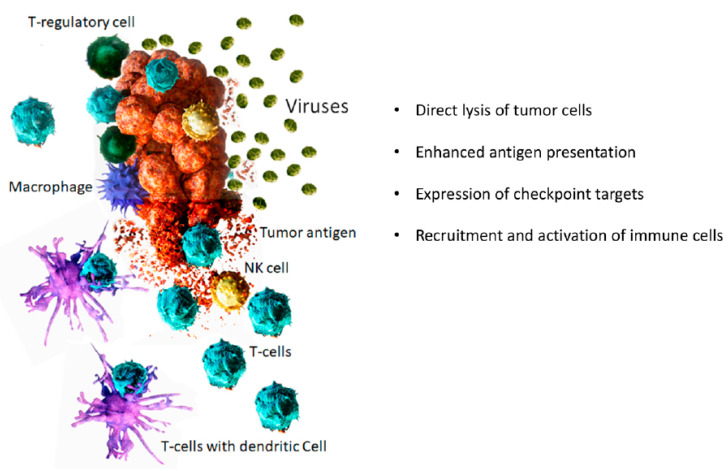
Mechanisms of tumor destruction by oncolytic viruses.

There has been an increasing interest in using viruses as potential therapeutics for different types of diseases. Two main ways in which viruses are being explored as therapeutics are (1) vectors for gene therapy and (2) oncolytic viruses. While viruses as vector for gene therapy are being studied for a wide range of diseases including cancer, “oncolytic viruses” are specific for cancer treatment. The major difference between viral vector for gene therapy and oncolytic virus is that viral vectors used in gene therapy are non-replicating viruses, whereas oncolytic viruses are replication-competent viruses. Oncolytic viruses (OV) are wild-type or engineered viruses that can selectively replicate in and kill cancer cells while leaving normal cells unharmed. OVs are a novel class of multi-mechanistic therapeutics for the treatment of cancer. Some of the mechanisms through which OVs exert their anti-cancer effect include direct lysis of cancer cells and activation of anti-tumor immunity (Figure 1).

The concept of using viruses to kill cancer is not new, there is anecdotal evidence of anti-tumor benefits from virus infections dating back to a century ago (reviewed in [1,2]). However, the field of oncolytic virus research has only recently gained momentum as a result of recent advancements in molecular biology and virology. In the last two decades, a wide diversity of virus families, including adenoviridae, poxviridae, herpesviridae, rhabdoviridae, reoviridae, paramyxoviridae, parvoviridae and picornaviridae, have been studied for their potential as oncolytic agents (reviewed in [3]). While the majority of them are still in preclinical stage of testing, some of them have completed different phases of clinical trials. Specifically, viruses belonging to the families adenoviridae, poxviridae, herpesviridae and reoviridae are the ones that have been studied in clinical settings. In this review article, we discuss the viruses from these four families that have been studied or are being studied in clinical trials.

## 2. Herpes Simplex Virus-1

Herpes simplex virus-1 (HSV-1) belongs to the family herpesviridae. It is an enveloped, double-stranded DNA virus with genome size of approximately 150 kbp that encodes ~89 genes [4]. Many HSV-1-based oncolytic viruses have been evaluated in clinical trials and most of them contain deletions in the neurovirulence gene *ICP34.5* [5]. Talimogene laherparepvec, a HSV-1, is the only oncolytic virus to have gained regulatory approvals in the Western world.

### 2.1. T-VEC

Talimogene laherparepvec (T-VEC) marketed as Imlygic, is a JS-1 strain of HSV-1 that was originally isolated from a cold sore [6,7]. The virus is attenuated through deletion of both copies of the neurovirulence gene infected cell protein 34.5 (*ICP34.5*). Furthermore, immunostimulatory properties of T-VEC have been improved by the deletion of the gene ICP47, an inhibitor of major histocompatibility complex I (MHC-I) mediated antigen presentation, and by the insertion of granulocyte-macrophage colony-stimulating factor (GM-CSF) expression cassettes at both loci of *ICP34.5*. The deletion of ICP47 enhances immune-mediated destruction of the virus-infected cells, whereas virus-encoded GM-CSF increases recruitment and activities of dendritic cells, ultimately boosting anti-tumor immunity [7].

In preclinical studies, T-VEC was found to induce strong cytotoxic activities against a wide range of tumor cell lines in vitro and caused tumor regression in murine models [8,9]. Followed by the promising preclinical results, T-VEC was tested for its safety in a Phase I clinical trial that included patients with refractory melanoma, breast cancer, gastric cancer or head and neck cancer [10]. In this trial, patients were primed with intratumoral injection of 10^6^ plaque forming units (PFUs) of virus followed by multiple injections of higher doses (10^7^ or 10^8^ PFUs). The treatment was well tolerated with the most common side effects being local inflammation and erythema febrile responses. Overall responses to the treatment were mild; however, it was encouraging to find that the virus was able to replicate in the tumors, produce GM-CSF, cause tumor necrosis and inflammation in both the injected and, in some cases, un-injected tumors. After this Phase I study, an initial Phase II clinical trial was performed that included 50 patients with stage IIIC unresectable or metastatic melanoma [11]. T-VEC was injected intratumorally at 10^8^ PFU at the interval of 2 weeks for up to 24 treatments. Tumor regression was observed not only in the injected lesions, but also in un-injected distant tumors including visceral metastases. The overall response rate (ORR) was 26% with 8 out of 13 responding patients experiencing a complete response. The treatment was well tolerated with the most common side-effects being inflammation at injection sites, flu-like symptoms and nausea. This encouraging Phase II trial was followed by a Phase III trial, which involved 436 patients with stage IIIB to IV melanoma. In this study, efficacy of intratumoral T-VEC treatment was compared with a subcutaneous injection of GM-CSF [12]. The ORR for T-VEC and GM-CSF was found to be 31.5%, and 6.4%, respectively. Also, 16.9% of patients treated with T-VEC experienced complete remission [13]. Following this Phase III study, T-VEC was approved by the FDA as monotherapy for unresectable melanoma following initial surgery.

Puzanov et al. reported the findings of a Phase Ib trial of T-VEC in combination with ipilimumab (a cytotoxic T-lymphocyte-associated antigen 4 (CTLA4) checkpoint inhibitor) in patients with advanced melanoma [14]. The combination therapy was found to be safe and the 18-month overall survival was 67%, which was greater than either T-VEC or ipilimumab monotherapy. Likewise, Ribas et al., reported the results of a Phase Ib study combining T-VEC with pembrolizumab (anti-PD-1 antibody) in patients with advanced melanoma [15]. The combination therapy was found to be well tolerated with the most common adverse events being fatigue, fever and chill, and no dose-limiting toxicities occurred. An objective response rate of 62% was achieved, with a complete response rate of 33% based on immune-related response criteria. The patients who responded to the combination therapy were those who had increased CD8+ T cells, elevated programmed death ligand 1 (PD-L1) expression and interferon gamma (IFN-y) gene expression after T-VEC treatment. Furthermore, there are more than 20 clinical studies currently recruiting patients (Clinicaltrials.gov; accessed on 1 March 2021) to determine safety and efficacy of T-VEC either as monotherapy or in combination with other therapeutics, including immune checkpoint inhibitors, in different types of cancers. Some of these studies, which are currently in trials, are mentioned in Table 1.

### 2.2. G207

G207 is another oncolytic HSV-1 that has completed several Phase I trials and is expected to begin a Phase II trial soon. Similar to T-VEC, G207 has deletions in both copies of *ICP34.5* genes. Additionally, G207 has the *ICP6* gene inactivated through the insertion of the *Escherichia coli lacZ gene*. The *ICP6* gene encodes a viral homolog of the cellular protein ribonucleotide reductase (RR), an enzyme required for the production of deoxy-ribonucleotides (dNTPs) [16]. Deletion of *ICP6* restricts virus replication to dividing cells with high levels of RR such as cancer cells. While this virus was originally designed for treatment of brain tumors, preclinical studies have found that the virus is effective against a wide range of cancer types including prostate, breast, head and neck, colon, ovaries, neuroblastoma, osteosarcoma and melanoma [17,18,19,20,21,22]. Results from the first clinical study of G207 were reported by Markert et al. [23]. A total of 21 patients with glioblastoma multiforme were enrolled in this study. The patients received 10^6^ to 3 × 10^9^ PFUs of the virus intratumorally. Some patients experienced adverse events, but toxicities were not directly related to the virus. Radiographic and neuropathologic analysis showed some evidence of anti-tumor activity. In another Phase I study, nine patients with recurrent malignant glioma were treated intratumorally with 10^9^ PFUs of G207 followed by treatment with 5 Gy radiation [24]. This combination treatment was well tolerated. In terms of efficacy, six of the nine patients had stable or partial response for at least one time point. A Phase II trial combining G207 with radiation therapy is expected to begin soon in pediatric patients with gliomas.

### 2.3. G47Δ

G47Δ is a variant of G207 that has also been tested for safety and efficacy in glioblastoma and prostate cancer in Japan. G47Δ has an additional deletion in the gene *ICP47*, which allows for enhanced MHC-I presentation on infected cells [22]. Interim analysis from a Phase II study in glioblastoma patient showed that the 1-year survival rate for G47Δ-treated patients (*n* = 13 patients) was significantly higher (92.3%) than the control group (15%) [25]. Severe adverse events (grade 2 fever) were observed in 2 out of 13 patients. Results of the Phase II study were published in late 2019 and this oncolytic virus has been designated as a “breakthrough therapy” drug by the Japanese government; thus, a fast track approval is expected [25].

### 2.4. ONCR-177

ONCR-177 is an oncolytic HSV-1 virus developed by Oncorus Inc. Unlike T-VEC and G207, ONCR-177 is not deleted in *ICP34.5* genes [26]. Target sequences for tissue-specific microRNAs (miRNA) are inserted into early genes (*ICP4*, *ICP27*, *UL8*), and in *ICP34.5,* that potently inhibit virus replication in normal cells, but not in cancer cells [26]. Furthermore, the virus contains mutations in the *UL37* gene that prevent axonal retrograde transport as well as latency, ensuring that virus will not damage neurons. Additionally, ONCR-177 encodes five immune-modulatory transgenes (IL-12, CCL4, FLT3LG, anti-CTLA4 and anti-PD-L1) driven by a dual bi-directional promoter [27]. ONCR-177 is currently in Phase I trial to determine the maximum tolerated dose and preliminary efficacy as monotherapy and in combination with pembrolizumab (NCT04348916).

### 2.5. RP1/2

RP1 is an oncolytic HSV-1 virus containing deletions in the neurovirulence genes ICP34.5 and ICP47. It has two transgenes inserted in its genome: GM-CSF and gibbon ape leukemia virus glycoprotein (GALV-GP R-), a fusogenic membrane glycoprotein. This virus is currently in Phase I trial as monotherapy for recurrent or advanced cutaneous squamous cell carcinoma (NCT04349436) or in combination with anti-PD-1 antibody (NCT04050436). RP1 has been further modified (known as RP2) to express anti-CTL4, which is also undergoing a Phase I trial (NCT03767348).

## 3. Vaccinia Virus

Vaccinia virus (VACV) is an enveloped virus belonging to the family poxviridae. VACV has a linear double-stranded DNA genome that is approximately 200 kpb in size and encodes ~200 genes [28]. VACV is one of the most intensively studied viruses for use in oncolytic therapy. VACV was used as a vaccine for the successful eradication of smallpox throughout the world, and thus, has a long-established safety profile in humans. Among many different oncolytic VACVs, Pexa-Vec is the most advanced one that has completed many clinical studies.

### 3.1. Pexa-Vec

Pexa-Vec (formerly JX-594) is a Wyeth strain of vaccinia virus that has *J2R*, encoding for thymidine kinase, replaced by the expression cassettes for human GM-CSF (hGM-CSF) and lacZ [29]. Pexa-Vec was deleted in *J2R* with the aim of increasing its cancer-specificity, and the hGM-CSF expression cassette was inserted with the aim of increasing anti-tumor immunity through the recruitment and activation of antigen-presenting cells (APCs) and cytotoxic T lymphocytes (CTLs) [29]. The construction of JX-594 and evaluation of its oncolytic properties was first reported by Kim et al. in 2006 [29]. In this study, the authors used two immune-competent orthotopic liver tumor models: a rabbit model with time-dependent metastases to lungs, and a carcinogen-induced rat liver tumor model [29]. While both rabbit and rat tumors support the replication of vaccinia virus, human GM-CSF is only partially active in rabbits and is inactive in rats. After the establishment of orthotopic tumors, rabbits were treated with 10^9^ PFUs of JX-594 intratumorally or intravenously. Compared to the control group, the intratumoral or intravenous injection of JX-594 potently inhibited the growth of primary tumors. Furthermore, while all control-treated rabbits developed lung metastases, none of the rabbits treated with JX-594 developed lung metastases. Interestingly, in the rat model in which human GM-CSF is not compatible, 10^8^ PFUs of JX-594 injected three times (intravenous) at the interval of 2 weeks, resulted in a complete tumor regression in majority of the treated mice. This study by Kim et al. was encouraging, especially because it involved immune-competent orthotopic tumor models, as opposed to other studies, most of which used human xenograft models and intratumoral injections for the evaluation of oncolytic viruses.

The prototype of JX-594 was used in a pilot Phase I trial in 1998 [30]. The virus used was a New York City Board of Health strain of VACV in which the *J2R* gene was replaced with expression cassettes for hGM-CSF and β-galactosidase. In this study, a total of seven patients with unresectable melanoma were treated twice weekly with intratumoral injections of escalating doses of the virus for 6 weeks. A maximum dose of 8 × 10^7^ PFU virus was used in the study and no maximum tolerable dose (MTD) was reached. Tumor regression in a fraction of patients was observed in this study, and systemic toxicity was limited to flu-like symptoms that resolved within 24 h. Followed by this pilot study, an open-label Phase I dose-escalation trial was conducted with substantially higher doses with the aim of defining safety and the MTD of JX-594 [31]. This study recruited 14 patients with refractory primary or metastatic hepatocellular carcinoma. Patients received intratumoral injection of the virus ranging in doses from 10^8^ PFU to 3 × 10^9^ PFU every 3 weeks. Grade 1–3 flu-like symptoms were observed in all patients and 4 out of 14 patients experienced transient grade 1–3 dose-related thrombocytopenia. Furthermore, patients treated with the highest dose (3 × 10^9^ PFU) displayed hyperbilirubinemia, which was considered dose-limiting. From this study, 1 × 10^9^ PFU was determined to be the MTD for JX-594. In addition to finding the MTD, several insights were gained from this Phase I trial. First, the study showed that it is possible for the virus to disseminate from injected to non-injected tumors through blood; virus replication and expression of GM-CSF were also confirmed. Second, tumor responses were observed in both injected and non-injected tumors. In terms of efficacy, out of 10 radiographically evaluable patients, 3 had partial response, 6 had stable disease and 1 had progressive disease.

Liu et al. reported another pilot study of JX-594 in advanced refractory hepatitis B virus (HBV)-associated hepatocellular carcinoma (HCC) [32]. The virus was injected intratumorally in a total of three patients, with the highest dose being 3 × 10^9^ PFU. In this study, virus treatment was found to be safe and anti-tumor efficacy was observed in all patients despite the presence of high levels of neutralizing antibodies. JX-594 was found to replicate in tumors and release of virus in circulation was observed. Additionally, this study showed that the virus treatment elevated the levels of anti-vascular cytokines, resulting in tumor vascular shutdown. Interestingly, JX-594 was also found to suppress the replication of hepatitis B virus in HCC patients. Another pilot study was reported by Heo et al. in 2011 on the combination of JX-594 with the kinase inhibitor, sorafenib, for the treatment of HCC [33]. In murine models, treatment of tumors with JX-549 followed by treatment with sorafenib resulted into superior anti-tumor efficacy than either agent alone. In human patients, the sequential treatment was well tolerated and associated with objective tumor responses.

In 2011, Breitbach et al. reported the findings from a Phase I dose-escalation trial of a single intravenous infusion of JX-594 [34]. The maximum dose used in the study was approximately 2 × 10^9^ PFU, which was generally well tolerated. This study included a total of 23 patients. The virus was detectable in tumor biopsies of patients who received virus at the dose ≥10^9^ PFU. This study provided the proof-of-concept regarding the feasibility of the systemic administration of JX-594 for the treatment of tumors.

Results from a Phase II dose-finding trial for JX-594 in liver cancer treatment were reported by Heo et al. in 2013 [35]. A total of 30 patients were enrolled in this study. Two doses of virus 10^8^ PFU (low-dose) and 10^9^ PFU (high-dose) were used for intratumoral infusions on day 1, 15 and 29 in patients with advanced hepatocellular carcinoma. The objective of this study was to compare the safety and efficacy of low- and high-dose treatments and induction of immunity against both cancer cells and the oncolytic virus. The objective response rate (15%) and intrahepatic disease control (50%) were found to be equivalent in the injected and non-injected distant tumors for both the doses. GM-CSF expression and the induction of anti-tumor immunity was observed at both the doses. While the tumor response rate and immune endpoints were similar for the low- and high-dose treatments, survival of patients treated with the high-dose treatment was significantly higher (median survival 14.1 months) than that for patients treated with the low-dose treatment (6.7 months).

Recently, results from a multicenter Phase IIb trial of Pexa-Vec in sorafenib-resistant hepatocellular carcinoma (TRAVERSE) were published by Moehler et al. [36]. Patients received best supportive care (BSC) alone or BSC plus Pexa-Vec. A single intravenous infusion (10^9^ PFU) was followed by up to five intratumoral injections. The treatment was generally well tolerated and evidence of immune induction against the virus as well as tumor antigens were observed. However, in this study Pexa-Vec failed to improve the survival of patients; the overall survival of patients treated with BSC alone (4.2 months) was not significantly different from the overall survival of patients treated with BSC plus Pexa-Vec. Currently, Pexa-Vec is being tested in combination with sorafenib in a worldwide Phase III PHOCUS trial in HCC patients.

Thus far, more than a dozen clinical trials have been completed with JX-594 (Pexa-Vec) in multiple types of malignancies, in which more that 400 patients have been injected with the virus. The MTD has been determined to be 10^9^ PFU. While all these studies have established an excellent safety profile for this virus, the anti-tumor efficacy of the virus remains uncertain.

### 3.2. GL-ONC1

GL-ONC1 (formerly known as GLV-1h68) is an oncolytic VACV based on the Lister strain developed by Genelux Corporation [37]. Like JX-594, GL-ONC1 is deleted in the *J2R* gene. Additionally, GL-ONC1 is deleted in the *F14.5L* and *A56R* genes. The virus encodes three transgenes: β-galactosidase, β-glucuronidase and Renilla luciferase–Aequorea green fluorescent protein [37]. In preclinical studies, the virus was found to be safe and efficacious against a variety of malignancies including colon cancer, breast cancer, glioma, ovarian cancer, pancreatic cancer and prostate cancer [38,39,40,41,42]. Followed by encouraging preclinical results, the virus has been evaluated in multiple early-stage clinical trials, where the virus was administered either intratumorally or intravenously. GL-ONC1 has been well tolerated in patients and no MTD was reached in any of the studies. While there has been some evidence of anti-tumor efficacies in early phase trials, further trials are needed to determine the overall anti-tumor efficacy of this virus.

### 3.3. vvDD

The vvDD (also called JX-929 or vvDD-CDSR) is a Western Reserve (WR) strain of VACV that is deleted in *J2R* and *C11R* (vaccinia growth factor) genes, and it encodes bacterial cytosine deaminase as well as the somatostatin receptor [43]. The WR strain of VACV is considered a laboratory strain that was never used as a vaccine. Although the WR strain is known to exert superior oncolytic activity compared to other strains of VACV, the WR strain has only been used in two clinical studies thus far [44]. Deletion of *J2R* and *C11R* allows for vvDD to selectively replicate in cells with an active E2F and epidermal growth factor receptor (EGFR/Ras) pathway, common features of many cancer cells [43,44,45]. The vvDD virus was found to exert potent anti-tumor efficacy against colon cancer, ovarian cancer and liver metastases in preclinical studies [43,44,45]. Followed by the encouraging preclinical results, a first-in-man study of vvDD was performed to determine the safety, systemic spread and anti-tumor efficacy in patients with solid tumors, and the findings of this study were reported in early 2015 by Zeh et al. [43]. In this dose-escalation study, up to 3 × 10^9^ PFU of virus, the maximum feasible dose, was injected intratumorally without dose-limiting toxicities. The virus was recovered from both injected and non-injected tumors. Although no clinical benefits were achieved in this study, it was encouraging to see that the WR strain of VACV was well tolerated even at the maximum feasible dose of 3 × 10^9^ PFU.

The second clinical study using vvDD was published by Downs-Canner et al. in 2016 [46]. In this Phase I study, vvDD was delivered intravenously in 11 patients with advanced colorectal or other solid tumors. A maximum of 3 × 10^9^ PFU virus was injected in patients. The virus was well tolerated with the most common adverse events being grade 1/2 flu-like symptoms. Tumor-specific replication of the virus was observed. Clinical benefits were minimal, but the authors concluded that excellent safety profile of the virus warrants further trials in combination with other therapeutics such as checkpoint inhibitors. Table 2 shows examples of VACVs in clinical trials.

## 4. Adenovirus

Adenoviruses are non-enveloped viruses with linear double-stranded DNA genome that ranges from 26 to 45 kbp in size [47]. A search on Clinicaltrials.gov for the term “oncolytic adenovirus” displayed a total of 45 studies in different phases of trials, out of which 11 have been completed and 14 are currently recruiting patients. An oncolytic adenovirus called H101 (also called Oncorine), is the first oncolytic virus to be approved for the treatment of cancer. H101 is an E1B-deleted adenovirus type 5 that was approved in China in November 2005 for the treatment of head and neck cancer [48]. Despite many trials in North America and Europe, oncolytic adenovirus has not been approved in these countries because the virus has failed to demonstrate meaningful anti-tumor efficacy. However, several investigators have developed modified strains of adenoviruses, which have shown greatly enhanced oncolytic potentials in preclinical studies. This has renewed the interest in pursuing adenovirus as an oncolytic agent.

### 4.1. DNX-2401

DNX-2401 (also known as Delta-24-RGD) is an oncolytic adenovirus in which the early gene E1A is inactivated through the deletion of 24 bp in the E1A gene [49]. E1A inactivation allows this virus to selectively replicate in cells with non-functional retinoblastoma protein, which is found in the majority of cancers [50]. Also, the fiber knob protein in this virus has been modified through the insertion of an arg-gly-asp (RGD) motif to target integrins on cancer cells as its primary receptor [49]. This virus has been studied mainly for the treatment of brain cancer. According to clinicaltrials.gov (accessed on 1 March 2021), DNX-2401 has completed four Phase I trials in patients with brain tumors/glioblastoma and one Phase I trial in patients with ovarian cancer and primary peritoneal cancer. In brain cancer patients, the virus has been studied as a single agent or in combination with other therapeutics such as interferon-y, temozolomide or pembrolizumab. While results from most of the studies have not been published, in 2019 Philbrick and Adamson published the findings of a Phase I study of DX-2401 in patients with recurrent high-grade gliomas [51]. In this study, 14 days after intratumoral injection of the virus, injected tumor was surgically removed from one group of patients but not from another group. The median overall survival (mOS) was 13.5 months in patients that underwent surgical resection, whereas the mOS was 9.5 months in patients that did not undergo surgical resection. The virus was well tolerated with no grade 3/4 adverse events. Currently there are two active trials listed for this virus: a Phase I trial in combination with conventional surgery and a Phase II trial in combination with pembrolizumab.

### 4.2. Enadenotucirev

Kuhn et al. used recombination and a directed evolution approach to create a chimeric group B oncolytic adenovirus called Enadenotucirev [52]. This virus is a chimera of adenovirus type 11 and adenovirus type 3, which was derived from a pool of seven different adenovirus serotypes used to infect human colorectal cancer cells HT-29. In preclinical studies the virus was found to demonstrate a 2-log increase in potency and selectivity in vitro when compared to one of the most studied oncolytic adenovirus ONYX-015. Furthermore, the virus was found to be much more potent than parental viruses and Onyx-015 in murine models and in human tumors ex vivo [52]. To date, three Phase I studies have been completed with this virus. Results from one of the Phase I studies were published by Machiels et al. in 2019. In this study, 61 patients with advanced epithelial tumors were enrolled and a maximum of 10^13^ virus particles were administered through intravenous infusion. The most common adverse events of grade 3 were hypoxia, lymphopenia and neutropenia, which were manageable [53]. There are currently two active Phase I studies with Enadenotucirev that are recruiting patients. The first one is recruiting patients with metastatic or advanced colorectal cancer, head and neck cancer or other epithelial tumors (NCT02636036). In this study, Enadenotucirev will be used in combination with nivolumab (PD-1 inhibitor). Another Phase I trial with Enadenotucirev is recruiting patients with locally advanced rectal cancer (NCT03916510), and patients will be treated with the virus in combination with radiotherapy and chemotherapy (capecitabine).

### 4.3. LOAd703

LOAd703 is another oncolytic adenovirus that has shown promising results in preclinical studies and is actively being tested in clinical studies for the treatment of a variety of cancers [54]. LOAd703 encodes two immune-stimulatory proteins: a trimerized CD40 ligand and a 4-1BB ligand. There are currently three active clinical trials involving LOAd703 that are recruiting patients for Phase I or II studies (Table 3). The first one is recruiting patients with pancreatic adenocarcinoma, ovarian cancer, biliary carcinoma and colorectal cancer (NCT03225989). In this Phase I study, patients will be treated with LOAd703 as monotherapy. The second one is recruiting patients with malignant melanoma, who will be treated with LOAd703 in combination with the checkpoint inhibitor atezolizumab in Phase I and II studies (NCT04123470). The third clinical study involving LOAd703 is currently recruiting pancreatic cancer patients, who will be treated with the virus in combination with gemcitabine, nab-paclitaxel and atezolizumab (NCT02705196).

### 4.4. ONCOS-102

Another oncolytic adenovirus being tested in clinical studies is ONCOS-102 (previously called CGTG-102). ONCOS-102 is a chimeric adenovirus constructed from adenovirus type 3 and 5, and is armed with the cytokine GM-CSF [55]. Thus far, the virus has completed one Phase I study and several more studies are currently recruiting patients for testing the safety and efficacy of this virus as a monotherapy or in combination with other therapies. In the first clinical study, 12 patients with advanced-stage solid tumors, refractory to available treatments, were recruited for a dose-escalation study [56]. Patients were treated intratumorally with multiple injections of virus doses ranging from 3 × 10^10^ to 3 × 10^11^ virus particles in combination with low-dose cyclophosphamide. No dose-limiting toxicity was observed, and hence, no MTD was identified in this study. In terms of anti-tumor efficacy, 4 out of 10 evaluable patients had stable disease at 3 months. Patients treated with the virus had a short-term elevation in pro-inflammatory cytokines and had an increase in tumor infiltration by lymphocytes, as determined by the immunohistochemistry of tumor biopsies before and after the treatment. In some patients, systemic induction of tumor-specific CD8+ T cells was observed. Furthermore, an upregulation in the levels of PD-L1 in the tumor was observed after virus treatment, suggesting that the efficacy of the virus could be enhanced by combining it with PD-L1 inhibitors. Hence, there are active clinical studies testing the safety and efficacy of ONCOS-102 in combination with PD-L1 inhibitors such as durvalumab (NCT02963831) and pembrolizumab (NCT03003676).

### 4.5. RNA Viruses

RNA viruses belonging to different families including coronaviridae, picornaviridae, reoviridae, retroviridae, rahabdoviridae and togaviridae have been studied for their oncolytic activities [57]. One common feature of RNA viruses is that they generate double-stranded RNA during their life-cycle, which causes the activation of cellular defense mechanisms, including the activation of protein kinase R (PKR) and the release of interferon [58]. However, majority of cancers have defective PKR signaling and interferon response pathways, making them naturally susceptible to RNA viruses. While many oncolytic RNA viruses have completed Phase I trials, the most advanced oncolytic RNA virus that has completed Phase I, II and III studies is reolysin (a reovirus). In this article we focus on reolysin as an example of oncolytic RNA virus.

## 5. Reovirus

Reoviruses are non-enveloped viruses with double-stranded, segmented RNA genome. The total genome size is 23.5 kbp, which is divided into 10 segments [59,60]. Wild-type reovirus preferentially replicates in cancer cells, ultimately leading to lysis of those cells [61]. Cancer selectivity of reovirus has been shown to be due to the overexpression of the oncogene *Ras* and the impairment in type-I interferon (IFN) signaling [62]. Reovirus is one of the most commonly studied viruses for its oncolytic usage. Reolysin (also known as Pelareorep), an unmodified reovirus type 3, Dearing strain [63], is the most advanced oncolytic reovirus that has completed multiple clinical trials. According to Clinicaltrials.gov (accessed in 1 November 2020), a total of 20 clinical trials have been completed with this virus (6 Phase I trials, 13 Phase II trials and 1 Phase III trial). The virus has been studied as a monotherapy or in combination with other therapeutics for the treatment of a multitude of cancers including metastatic breast cancer, advanced-stage head and neck cancer, metastatic ovarian cancer, malignant gliomas, prostate cancer and metastatic melanoma. Out of the total 20 completed trials, Reolysin was used as monotherapy in 5 studies, and in the remaining 15 studies it was combined with chemotherapeutics such as gemcitabine, irinotecan, docetaxel, carboplatin, paclitaxel and cyclophosphamide (Table 3). Currently, there are five clinical trials on Reolysin which are active and recruiting patients.

### 5.1. Reolysin as Monotherapy

Results of a Phase I trial of Reolysin were published in 2013 by Morris et al. [64]. In this study, a total of 19 patients with advanced-stage solid tumors including breast cancer, head and neck cancer and melanoma were enrolled. Up to 1 × 10^10^ PFU virus was injected intratumorally and patients were monitored for safety and efficacy. Treatment was well tolerated with the most common toxicities being grade 2 local erythema and transient flu-like symptoms. All patients showed increasing levels of antibody titers against the virus. In terms of efficacy, 7 out of 19 patients demonstrated some level of anti-tumor response in the injected tumors: 1 had complete response, 2 had partial response and 4 had stable disease.

Vidal et al. reported the first clinical study on intravenous delivery of Reolysin [65]. In this Phase I study, a total of 33 patients with advanced cancer were treated intravenously with escalating doses of the virus, up to 3 × 10^10^ tissue culture infective dose (TCID50) for five consecutive days every 4 weeks. Grade 1 and 2 toxicities included fever, fatigue and headaches, which were independent of the virus doses. Virus-neutralizing antibodies were developed in all patients, with the peak titer detected 4 weeks after the first injection. Evidence of virus localization and replication in tumor was observed in three patients. The study report did not mention anything about anti-tumor efficacy, perhaps because no anti-tumor efficacy was observed.

Gollamudi et al. reported results from another clinical study involving intravenous administration of Reolysin [66]. In this dose-escalation study, 18 patients with advanced-stage solid tumors were treated with Reolysin intravenously every 4 weeks in doses ranging from 1 × 10^8^ to 3 × 10^9^ TCID50. Dose-limiting toxicity was not reached, and the common treatment-related toxicities included fatigue and fever. A virus-neutralizing antibody was developed in all patients during the course of treatment. Furthermore, virus shedding was observed in 6 out of 18 patients, and the patients showing virus shedding appeared to be more responsive to the treatment. In this study, the overall clinical benefit rate was 45%: one patient experienced partial response and seven attained stable disease.

The first Phase II trial for intravenous monotherapy of Reolysin was initiated in 2008 [67]. In this study, 21 patients with metastatic melanoma were treated intravenously with 3 × 10^10^ TCID50 on days 1–5 of each 4 week cycle. Similar to previous Phase I trials, the treatment was well tolerated. Productive reovirus replication was observed in the tumor biopsies of two patients. Unfortunately, no objective response was observed in this study. Likewise, results from another Phase II trial were reported by Mite et al. (NCT00503295). In this study, 43 patients with bone and soft tissue sarcoma metastatic to the lung were recruited. Patients were treated intravenously with Reolysin every 28 days. Toxicities were mild to moderate, including fever, chills, fatigue, diarrhea, cough, dyspnea, neutropenia and thrombocytopenia. Overall, 14 patients experienced stable disease for more than 2 months, and 5 patients had stable disease for more than 6 months.

### 5.2. Reolysin in Combination with Other Therapies

While clinical studies with Reolysin as monotherapy have demonstrated that the virus is safe, the lackluster efficacy of the virus as monotherapy has prompted investigators to combine it with other therapeutics. Over a dozen clinical studies encompassing different phases of trials have been completed so far with Reolysin in combination with radiation, chemo- or biological therapies. Harrigton et al. reported the findings of a Phase I trial combining reovirus in patients receiving palliative radiotherapy [68]. In total, 23 patients with various solid tumors of advanced stages were treated in two stages. In the first stage, patients received 20 Gy of radiation followed by two intratumoral injections of reovirus at doses between 1 × 10^8^ and 1 × 10^10^ TCID50. In the second stage, patients received a higher dose of radiation (36 Gy) followed by 2, 4 or 6 doses of 1 × 10^10^ TCID50 virus. The combination treatments were found to be safe with mild (grade 1 to 2) toxicities. Two of seven evaluable patients in the low-dose radiation group showed partial response, and five patients had stable disease. Likewise, in the high-dose radiation treatment, five of seven evaluable patients experienced partial response and two had stable disease. No virus shedding was detected in blood, urine, stool and sputum of the patients. Given the excellent safety profile and better efficacy, compared to previous studies using reovirus as monotherapy, the authors concluded that this combination should be evaluated in newly diagnosed patients receiving radiation with curative intent.

Lolkema et al. reported results of the first clinical study combining reovirus with a chemotherapy [69]. In this Phase I study, 16 patients with advanced-stage cancers receiving gemcitabine were treated with a single dose of up to 3 × 10^10^ TCID50 reovirus. Overall, 3 out of 16 patients experienced dose-limiting toxicity, with 2 patients having liver enzymes increased to grade 3, and 1 patient with levels of troponin elevated to grade 3. The increase in liver enzymes was found to be transient and reversible. In this combination treatment, neutralizing-antibody responses to reovirus were delayed and attenuated. Following this study, a Phase II study was initiated to evaluate the safety and efficacy of reovirus in combination with gemcitabine in patients with metastatic pancreatic cancer (NTC00998322). As of today, this study has been completed but findings of the study have not been published. A variety of other chemotherapeutics including carboplatin, irinotecan, cyclophosphamide, paclitaxel and docetaxel have been studied in combination with Reolysin in Phase I and/or Phase II trials against a multitude of cancers. Finally, a Phase III trial of Reolysin in combination with carboplatin and paclitaxel has been completed in patients with head and neck cancer. Findings of the study have not been reported yet.

Reolysin has also been studied in combination with other biological therapeutics. The first biological therapeutic to be combined with Reolysin was bevacizumab, a monoclonal antibody against vascular endothelial growth factor. In a Phase I trial, colorectal cancer patients with mutant KRAS were treated with Reolysin and bevacizumab together with the chemotherapeutics irinotecan, leucovorin and 5-fluorouracil (NCT01274624). The study has been completed, but the results have not been published yet. Additionally, several clinical studies combining reovirus with checkpoint inhibitors (CKIs) are currently underway (Table 4).

## 6. Conclusions and Perspectives

The field of oncolytic virotherapy is rapidly growing, with thousands of preclinical studies reported, and over 100 clinical trials performed thus far. The approval of T-VEC by the FDA in 2015 amplified the interest of cancer researchers and clinicians in this novel therapeutic approach. Apart from T-VEC, there are many oncolytic viruses that have completed different phases of clinical studies. From all the clinical trials performed so far, oncolytic viruses appear to be safe at maximum feasible doses, and in most cases, the maximum tolerable doses have not been reached. While OVs have shown excellent safety profiles, their anti-tumor efficacies are modest at best. Nevertheless, for most viruses, the MTD has not been reached, and it is likely that their anti-tumor efficacies could be increased with future advancements in virus manufacturing technology. Alternatively, novel viruses with higher anti-tumor potency could be generated, which may allow researchers to achieve maximum anti-tumor benefits at doses that are feasible with the current manufacturing process. For example, our group created a chimeric poxvirus through recombination among nine poxviruses, encompassing different strains of VACV and different species of poxvirus [70]. The chimeric poxvirus (called CF33) was found to be more potent than the parental viruses in killing cancer cells in vitro. Furthermore, the CF33 was found to abrogate tumor growth in mice at doses much lower than the doses of other oncolytic poxviruses reported in the literature [70,71,72]. We are currently in the process of obtaining regulatory approvals to initiate a Phase I trial with this virus.

Most of clinical trials, including those for T-VEC, have used intratumoral route for virus administration. The reason for using intratumoral administration is to avoid virus neutralization by antibodies and virus sequestration by macrophages, which could severely impede OVs, especially during repeated injections. The downside of the intratumoral route for virus injection is that the virus could not be administered in tumors that are inaccessible for direct injections. Furthermore, once a cancer has been metastasized, it is not possible to find all the metastatic nodules and inject them. While some studies have shown that injection of virus in one tumor can lead to regression of distant un-injected tumors, this phenomenon of abscopal effect has been observed only in small subset of patients. Recently, some clinical studies have used the systemic route for the delivery of oncolytic viruses, showing the feasibility of systemic injections of OVs in patients. The anti-tumor efficacy achieved through systemic injections of viruses has been modest, just as is the case for intratumoral injections of OVs.

Cancer is a complex disease that often requires a complex treatment comprising a combination of several therapeutic approaches. Results from clinical trials suggest that oncolytic viruses as monotherapy are less likely to achieve optimum therapeutic benefits. However, oncolytic viruses appear to be good candidates for combination with other therapeutic approaches, especially immunotherapy. Oncolytic viruses have been shown to subvert immune-suppressive microenvironments in tumors, making them conducive for the effectiveness of immunotherapeutics (reviewed in [73]). A number of preclinical studies have shown the effective combination of oncolytic viruses with immunotherapeutics. Consequently, recent clinical trials with oncolytic viruses have been heavily focused on the combination of viruses with immunotherapeutics, especially with immune checkpoint inhibitors. While it is too early to conclude if the impressive results from preclinical studies combining oncolytic viruses with immunotherapeutics could be translated in the clinical studies, there are some indications that the combination will work in clinic. Ribas et al. reported the results of a Phase Ib trial combining T-VEC with pembrolizumab (anti-PD-1 antibody) in patients with metastatic melanoma [15]. The overall response rate (62%) and complete response rate (33%) achieved by the combination treatment were almost double the ORR (31.5%) and CR (16.9%) achieved by T-VEC alone in the OPTIM (Phase III) trial. Results from other clinical studies combining oncolytic viruses with immunotherapeutics are expected to be published soon.

In summary, the field of oncolytic virotherapy has made dramatic strides in the last two decades, culminating in the approval of the first OV in the Western world. With many oncolytic viruses having completed Phase II trials, it is likely that more oncolytic viruses, perhaps in combination with immunotherapeutics, will soon receive approval from regulatory agencies for the treatment of different malignancies.

## Figures and Tables

**Table 1:** Examples of oncolytic herpes simplex viruses (HSVs) currently under clinical trials.

Virus	Transgene	Combination	Tumor Type	Phase	Reference
G207	None	Radiation	Brain tumor	II	NCT04482933
ONCR-177	IL-12, CCL4, FLT3LG, αCTLA4 and αPD-1	pembrolizumab	Melanoma and other solid tumors	I	NCT04348916
OH2 (HSV-2)	GM-CSF	Irinotecan or HX008	Gastrointestinal tumors and other solid tumors	I & II	NCT03866525
RP1	GALV-GP and GM-CSF	None	Cutaneous squamous cell carcinoma	IB	NCT04349436
RP1	GALV-GP and GM-CSF	Cemiplimab	Cutaneous squamous cell carcinoma	I	NCT04050436
RP2	GALV-GP and GM-CSF	Nivolumab	Advanced solid tumors	i	NCT03767348
T-VEC	GM-CSF	Ipilimumab and Nivolumab	Breast Cancer	I	NCT04185311
T-VEC	GM-CSF	None	Angiosarcoma of skin	II	NCT03921073
T-VEC	GM-CSF	Pembrolizumab	Sarcoma	II	NCT03069378
T-VEC	GM-CSF	Pembrolizumab	Cutaneous melanoma	II	NCT03842943

**Table 2:** Examples of oncolytic vaccinia viruses (VACVs) in clinical trials.

Virus	Transgene	Combination	Tumor Type	Phase	Status	Reference
Pexa-Vec	GM-CSF	REGN2810 (anti-PD-1)	Renal cell carcinoma	I	Recruiting	NCT03294083
Pexa-Vec	GM-CSF	Ipilimumab	Any except liver cancer	I	Recruiting	NCT02977156
Pexa-Vec	GM-CSF	Cyclophosphamide and Avelumab	Solid tumors and soft tissue sarcoma	I & II	Recruiting	NCT02630368
Pexa-Vec	GM-CSF	Sorafenib	Hepatocellular carcinoma	II	Completed	NCT01171651
Pexa-Vec	GM-CSF	None	Hepatocellular carcinoma	II	Completed	NCT01636284
Pexa-Vec	GM-CSF	BSC	Hepatocellular carcinoma	II	Completed	NCT01387555
T601	FCU1	5-Fluorocytosine	Solid tumors	I & II	Recruiting	NCT04226066
TBio-6517	FLT3L, IL-12, αCTLA-4	Pembrolizumab	Solid Tumors	I & II	Recruiting	NCT04301011
GL-ONC1	Luc-GFP, β-Galactosidase β-glucuronidase	Bevacizumab	Ovarian cancer, peritoneal carcinomatosis and cancer of fallopian tube	I & II	Recruiting	NCT02759588
GL-ONC1	Luc-GFP, β-Galactosidase β-glucuronidase	None	Head and neck cancer	I	Completed	NCT01584284
GL-ONC1	Luc-GFP, β-Galactosidase β-glucuronidase	None	Solid tumors	I	Completed	NCT00794131
vvDD	Cytosine deaminase and somatostatin receptor	None	Solid tumors	I	Completed	NCT00574977

**Table 3:** Examples of clinical trials with adenoviruses.

Virus	Transgene	Combination	Tumor Type	Phase	Status	Reference
LOAd703	CD40L, 4-1BBL	None	Pancreatic cancer, ovarian cancer, biliary carcinoma, colorectal cancer	1 & 2	Recruiting	NCT03225989
LOAd703	CD40L, 4-1BBL	atezolizumab	Malignant Melanoma	1 & 2	Recruiting	NCT04123470
LOAd703	CD40L, 4-1BBL	Gemcitabine, nab-paclitaxel and atezolizumab	Pancreatic cancer	1 & 2	Recruiting	NCT02705196
TILT-123	hIL-2, TNFa	None	Metaststic melanoma	1	Recruiting	NCT04217473
DNX-2440	OX40L	None	Glioblastoma	1	Recruiting	NCT03714334
CG0070	GM-CSF	None	Bladder cancer	2	Completed	NCT02365818
Delta-24-RGD	None	None	Brain tumor	1 & 2	Completed	NCT01582516
MG1-MAGEA3	MAGEA3	Pembrolizumab, Ad-MAGEA3	NSCLC	1 & 2	Completed	NCT02879760

**Table 4:** Examples of clinical trials with Reolysin.

Combination	Tumor Type	Phase	Status	Reference
Paclitaxel	Ovarian cancer, peritoneal carcinomatosis and cancer of fallopian tube	2	Completed	NCT01199263
None	Sarcoma	2	Completed	NCT00503295
Carboplatin Paclitaxel	NSCLC	2	Completed	NCT00861627
Irinotecan, Leucovorin, 5-FU, Bevacizumab	Colorectal cancer	1	Completed	NCT01274624
Carboplatin Paclitaxel	Head and Neck cancer	3	Completed	NCT01166542
None	Malignant glioma	1	Completed	NCT00528684
Carboplatin Paclitaxel	Lung Cancer	2	Completed	NCT00998192
Carboplatin Paclitaxel	Head and neck cancer	2	Completed	NCT00753038
Carboplatin Paclitaxel	Metastatic melanoma	2	Completed	NCT00984464
Paclitaxel, Avelumab	Metastatic breast cancer	2	Recruiting	NCT04215146
Carfilzomib, Dexamethasone, Nivolumab	Recurrent Plasma cell myeloma	1	Recruiting	NCT03605719
Retifanlimab	TNBC	2	Recruiting	NCT04445844
